# Intercropping Cover Crops for a Vital Ecosystem Service: A Review of the Biocontrol of Insect Pests in Tea Agroecosystems

**DOI:** 10.3390/plants12122361

**Published:** 2023-06-18

**Authors:** Sabin Saurav Pokharel, Han Yu, Wanping Fang, Megha N. Parajulee, Fajun Chen

**Affiliations:** 1 Department of Entomology, College of Plant Protection, Nanjing Agricultural University, Nanjing 210095, China; pokharelsabin93@gmail.com; 2 Department of Forest Genetics and Breeding, College of Forestry, Nanjing Forestry University, Nanjing 210037, China; yuhan00107@163.com; 3 Department of Tea Science, College of Horticulture, Nanjing Agricultural University, Nanjing 210095, China; fangwp@njau.edu.cn; 4 Texas A&M AgriLife Research and Extension Center, 1102 East Drew Street, Lubbock, TX 79403, USA; megha.parajulee@ag.tamu.edu

**Keywords:** tea agroecosystem, cover crops, ecosystem services, biological control, natural enemies, environmentally benign, sustainable pest management

## Abstract

The intercropping of cover crops has been adopted in several agroecosystems, including tea agroecosystems, which promotes ecological intensification. Prior studies have shown that growing cover crops in tea plantations provided different ecological services, including the biocontrol of pests. Cover crops enrich soil nutrients, reduce soil erosion, suppress weeds and insect pests, and increase the abundance of natural enemies (predators and parasitoids). We have reviewed the potential cover crops that can be incorporated into the tea agroecosystem, particularly emphasizing the ecological services of cover crops in pest control. Cover crops were categorized into cereals (buckwheat, sorghum), legumes (guar, cowpea, tephrosia, hairy indigo, and sunn hemp), aromatic plants (lavender, marigold, basil, and semen cassiae), and others (maize, mountain pepper, white clover, round-leaf cassia, and creeping indigo). Legumes and aromatic plants are the most potent cover crop species that can be intercropped in monoculture tea plantations due to their exceptional benefits. These cover crop species improve crop diversity and help with atmospheric nitrogen fixation, including with the emission of functional plant volatiles, which enhances the diversity and abundance of natural enemies, thereby assisting in the biocontrol of tea insect pests. The vital ecological services rendered by cover crops to monoculture tea plantations, including regarding the prevalent natural enemies and their pivotal role in the biocontrol of insect pests in the tea plantation, have also been reviewed. Climate-resilient crops (sorghum, cowpea) and volatile blends emitting aromatic plants (semen cassiae, marigold, flemingia) are recommended as cover crops that can be intercropped in tea plantations. These recommended cover crop species attract diverse natural enemies and suppress major tea pests (tea green leaf hopper, white flies, tea aphids, and mirid bugs). It is presumed that the incorporation of cover crops within the rows of tea plantations will be a promising strategy for mitigating pest attacks via the conservation biological control, thereby increasing tea yield and conserving agrobiodiversity. Furthermore, a cropping system with intercropped cover crop species would be environmentally benign and offer the opportunity to increase natural enemy abundance, delaying pest colonization and/or preventing pest outbreaks for pest management sustainability.

## 1. Introduction

Tea, *Camellia sinensis* (L.) Kuntze, is an important economic crop used to produce the world’s oldest and most popular caffeine-containing beverage of immense cultural and medicinal significance [1,2]. Tea farmers have adopted different agricultural management practices (AMPs) for ecological intensification, such as diverse agroforestry cropping systems [3,4], crop rotation [5], contour farming [6], and the planting of shade trees [7]. Among these AMPs, the use of cover crops has been a proven agricultural practice for ecological intensification [8]. Cover crops are the crucial components of a sustainable crop production system because of their critical ecological services. Cover crops grown between rows of primary crops provide more benefits than conventional cropping systems, where ecosystem services are strengthened by lessening anthropogenic inputs [9,10]. Ecosystem services such as erosion control, water quality management, soil moisture retention, the accumulation of soil organic matter (SOM), and the regulation of greenhouse gas emissions, weeds, disease, and pest control, are intensified by cover crops [11]. Moreover, incorporating diverse cover crops helps with biological pest suppression due to improved natural enemy abundance and diversity [12]. Cover cropping is an essential biodiversity-based strategy that provides a compatible as well as a flexible solution for sustainable agriculture by maximizing beneficial effects on biodiversity and ecosystem services [12,13,14]. In addition to providing food resources, cover crops provide habitats, shelter, and desirable microclimates for natural enemies, which helps to deliver biological control services [15,16]. The most common intercropping system being adopted globally is cereal–legume intercropping [17,18]. The intercropping of leguminous cover crops can decrease N-fertilizer requirements by minimizing the potential for short-term N-immobilization and biological N-fixation [19,20]. And the intercropping of non-leguminous cover crops has been attributed to a higher Carbon (C)/Nitrogen (N) ratio (>25), with increased amounts of lignin, a high-carbon complex organic material within plant cell walls [20]. 

**Figure 1:**
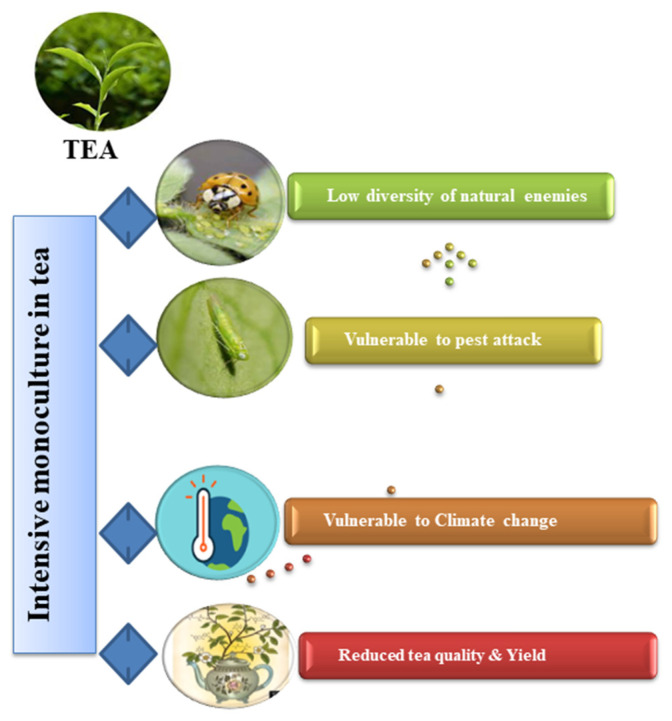
Effects of intensive monoculture in the tea plantations.

Growing a cash crop with a cover crop benefits the primary crop or the whole farming system [21,22]. Investing in agro-ecological strategies is an effective way of building climate resilience in tea production systems, as they support both environmental and human well-being without high-cost input materials, thereby alleviating pest attacks and soil nitrogen pressure [23,24]. Intensive monocultures in tea have given rise to numerous severe problems in the agroecosystem (as seen in Figure 1). Intercropping cover crops appears to be a promising alternative strategy for maximizing ecosystem services in the biocontrol of tea pests. Selecting appropriate cover crop species and identifying agro-ecological practices beneficial to biological pest control services are essential for successful cover cropping [12,25]. Cover crops that do not intensively compete with tea plants for resources (land, light, and water), require less nitrogen, deter pests, and enhance natural enemies are of utmost importance. Legume covers, which form symbiotic relationships with soil rhizobia and fix atmospheric nitrogen, are the most potent cover crops. Aromatic plant species produce blend volatiles as secondary metabolites in tea plantations and help to prevent the colonization of significant tea pests. Thus, legumes and aromatic plants integrated into the intercropping system provide better control of tea arthropod pests with less chemical insecticide usage and minimal environmental impact [26,27]. While reviewing the literature, it was evident that systematic information on the role and scope of cover crops in the tea agroecosystem and their ecological services in the biocontrol of tea pests is lacking. Thus, the current review aims at synthesizing the benefits that cover crops intercropped in tea plantations can provide regarding ecosystem service delivery and the biocontrol of pests. Additionally, we reviewed potential cover crops that can be intercropped in the tea agroecosystem, and which can provide effective ecological services in changing climates. 

## 2. Cover Cropping in Tea Agroecosystems

Several prior studies have indicated the importance of intercropping cover crops (legumes, cereal crops, and aromatic plants) in the tea agroecosystem. Here, we analyzed cover crops commonly cultivated in different intercropping systems, including the tea agroecosystem. We explored the potentialities of diverse cover crops suitable for tea plantations based on their ecosystem services delivery in pest control (Table 1). Potential cover crops that can be intercropped in tea agroecosystem based on their ecosystem services delivery on pest control attributes were grouped into cereals (buckwheat, sorghum), legumes (guar, cowpea, tephrosia, hairy indigo, and sunn hemp), and aromatic plants (lavender, marigold, basil, and semen cassiae). Potential cover crops with promising biocontrol prospects are maize, mountain pepper, white clover, round-leaf cassia, and creeping indigo. We compared the significance of different cover crop species that have been used or have the potential to be intercropped in the tea agroecosystem. Moreover, diverse crop species augment the biocontrol of pests in the tea agroecosystem by increasing the abundance of natural enemies. 

## 3. Biological Control: An Effective Ecosystem Service of Cover Crops

Biological control, an essential ecosystem service, is adopted in an agroecosystem with the objective of sustainable pest control. There are three general approaches to biological control: classical, augmentative, and conservation. Conservation biological control (CBC) is a scientific strategy for enhancing pest control by conserving natural enemies in agroecosystems [53]. Cover crops might fuel conservation biological control by preserving and maintaining natural enemies in the tea agroecosystem. The tea agroecosystem is rich in beneficial and phytophagous arthropods, which are active and abundant throughout the year [54]. The excessive application of pesticides in monoculture tea plantations not only destroys beneficial arthropods but also dismantles biodiversity [55]. According to Ozawa [56], a destructive insect pest, the tea green leafhopper (*E.onukii* Matsuda), has developed resistance to some intensively used insecticides (imidacloprid or related neonicotinoids) in Japanese tea gardens. Therefore, sustainable pest management strategies such as biological control offer an exciting alternative for controlling these notorious tea insect pests [57]. Biocontrol in tea monoculture encourages agro-biodiversity conservation by reconciling new agricultural habitats and enhanced natural enemies [58,59]. 

Intercropping cover crops enhance soil organic matters, which favors the enhanced diversity of natural enemies effective in pest suppression [60]. Intercropping ground cover promotes an essential ecosystem service, i.e., biological control, where pest density decreases with augmented natural enemies [43,61]. The enhanced predator abundance in intercropped tea plantations can effectively improve the anti-interference capability of the whole tea plantation ecological system [16,62,63]. A stable insect community structure with an increased number of natural enemies (predatory beetles and mites) was observed in the tea plantations intercropped with cover crops as compared to the pure tea plantation [64,65]. Hence, the dominant role of intercropping cover crops in enhancing beneficial arthropod biodiversity paves the pathway for conservation biological control in tea plantations [43]. The pivotal functions of intercrops in conservation biological control cannot be underestimated because of their substantive roles in attracting and sustaining natural enemies, besides providing food and shelter [16,53]. The habitat management approach is key for maintaining arthropod biodiversity and natural arthropod enemies in tea plantations, which could indirectly assist in the biological control of insect pests [16,64]. Intercropping cover crops in tea agroecosystem promotes habitat manipulation and enhances the diversity of beneficial arthropods, thereby delivering a critical ecosystem service; pest regulation via conservation biocontrol. Ecosystem services delivered by the natural enemies and predators prevalent in the tea agroecosystem are discussed in detail in the later sections focusing on cover crop species and the natural enemies. 

### 3.1. Biological Control and the Increased Diversity of Natural Enemies 

**Figure 2:**
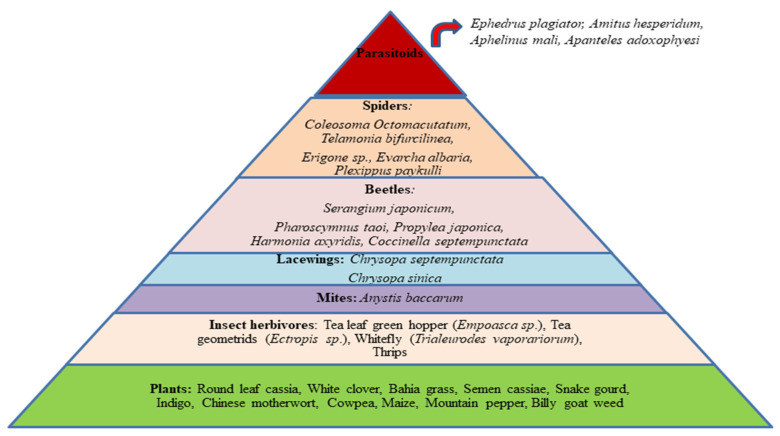
Trophic groups’ interactions among cover crops, insect herbivores, and natural enemies (predators and parasitoids) in the tea agroecosystem.

Intercropping tea plants with cover crops seems a promising ecological model to revitalize the agroecosystem, where the trophic interaction between natural enemies and tea pests helps to reduce the pest attack (seen in Figure 2). This ecological model might lower the abundance of economically important insect pests in tea plantations via conservation biological control. Choosing appropriate cover crops leads to a lower abundance of leafhoppers, a higher population abundance, and a prominent species richness of predators or parasitoids in tea plantations [52]. Legumes and cereal crops have been commonly used as cover crops in tea plantations. However, recently, aromatic plants (lavender, flemingia, cassia, mint, Chinese motherwort, semen cassiae) and climate-resilient crops (sorghum and cowpea) have also emerged as effective cover crops. These crops mentioned above might deliver an effective ecosystem service; the biocontrol of insect pests.

The incorporation of aromatic plant species in tea plantations mitigated pest attacks, enhanced the populations of natural enemies, and hence improved conservation biological control [66]. Intercrops (*Cassia tora* L. and *Chamaecrista rotundifolia* Greene) in tea plantations increased the abundance of arthropod predators by providing feasible microhabitats for nests and shelter [43,52]. Intercropping tea with the aromatic plant *Cassia tora* L. helped control the critical phytophagous pest (*E. vitis*), assisted by the biocontrol approach. The populations of generalist predators (ladybird beetles and lacewings) increased due to the release of repellent volatiles [52]. Three prime volatiles that exerted repellent effects on leafhoppers were p-cymene, limonene, and 1,8-cineole [52]. Also, tea intercropped with *C. tora* showed an increased abundance of the natural enemy (dragonfly) *Sympetrum croceolum* Selys 1883 [52]. Zhang et al. [66] mentioned that intercropping tea with aromatic plants, *Cassia tora* L., *Leonurus* artemisia Loureiro and Mentha haplocalyx Briq, reduced the infestation of *E. onukii* Matsuda in tea plantations. Intercropping cover crops (*Lavandula pinnata* Lundmark, *Cassia tora* L., *Hedyotis uncinella* Hook, *Trifolium repens* L., *Vigna sinensis* L.) in the tea plantation helped in reducing the pest attack of TGL (*E.vitis* Gothe) with the increase in the diversity of generalist predators and parasitoids [67,68,69]. The population abundances of tea geometrid moth caterpillars (*Ectropis oblique* Prout and *Ectropis griseescens* Warren) decreased with the intercropping of cover crops (*Chamaecrista rotundifolia* Greene and *Indigofera hendecaphylla* Jacq.) in tea. The population of tea geometrids decreased due to the increased abundance of predatory parasitoids, namely, Formicidae, Araneida, Mymaridae, Braconidae, and Trichogrammatidae [43]. Tea plants intercropped with aromatic plants (*Ocimum basilicum* L. and *Perilla frutescens* L.) decreased the abundance of crucial tea pests: *Empoasca onukii* Matsuda and *Apolygus lucorum* Meyer-Dür. The reduction in the pest attack of tea green leaf hopper and mirid bug was due to the increased diversity of natural enemies and predators (coccinellids, lacewings, parasitoids, and spiders). Ye et al. [70] mentioned that intercropping tea plants with economic crops (citrus, waxberry, and snake gourd) helped suppress key tea pests by increasing the diversity and species richness of key araneid natural enemies. Intercropping tea with *Ageratum conyzoides* assists in the biological control of the tea pest *Empoasca pirisuga* Matumura by increasing the diversity of predatory mites (*Anystis baccarum* L.) [71]. White clover (*Trifolium repens* L.) as an intercrop in tea reduced the populations of key tea pests: tea geometrid (*Ectropis grisescens* Warren), tea green leaf hopper (*E. vitis*), and tea aphid (*Toxoptera aurantii* Boyer de Fonscolombe) [67]. Moreover, the population of natural enemies (Araneae, coleoptera, hymenoptera) in the tea ecosystem increased [67]. Maize as an intercrop in tea plantations reduced the population of tea green leaf hoppers (*E. onukii* Matsuda) and white flies (*Trialeurodes vaporariorum* Westwood) [62].

The predominant natural enemies on tea plantations are ladybird beetles, spiders, assassin bugs, lacewings, and praying mantises [72]. The potential of intercropping to enhance arthropod biodiversity provides a gateway for biological pest control in tea plantations [43]. The most abundant natural enemies (predators and parasitoids) in the tea plantations after intercropping cover crops (as seen in Figure 2) and their ecosystem services in biological control are discussed below.

#### 3.1.1. Ladybird Beetles

Coleopterans are distinct members of food webs in tea plantations, with high species richness and abundance [65]. Tea plants intercropped with herbaceous perennial legumes (*Chamaecrista rotundifolia* Greene and *Trifolium repens* L.) had high beetle abundance and species richness, while tea plants intercropped with *Paspalum notatum* Flugge had enhanced biomass and species richness [65,67]. The most abundant predatory beetles were *Serangium japonicum Chapin* and *Pharoscymnus taoi* Sasaji in the tea plantations intercropped with C. rotundifolia and *P. notatum* [65]. *Propylea japonica* Thunberg, *Harmonia axyridis* Pallas, and *Coccinella septempunctata* L. were the predominant species of coccinellids in the tea plantations intercropped with an aromatic plant (*Cassia tora* L.) [66]. Cover crops (round leaf cassia, white clover, cassia) intercropped in tea plantations provided a feasible habitat and food source for the ladybird beetles, which helped reduce the attack of tea pests (*A. lucorum* and *E. onukii*). 

#### 3.1.2. Spiders

Spiders are predators of insect pests in cultivated ecosystems, and the maintenance of a thick layer of leaves within the tea canopy is essential for sustaining spider communities [73]. Intercropping cover crops can profoundly affect the spider communities in tea plantations due to the favorable habitat and food source provided by diverse cover crops. The Araneida number was high on the plots where tea was intercropped with *C. rotundifolia* and *I. hendecaphylla*, where they helped to check the population of *Empoasca onukii* Matsuda, Thysanoptera, and Geometridae caterpillars [43]. 

A well-maintained tea canopy, where *C. rotundifolia* and *P. notatum* were used as cover crops, saw *Coleosoma octomacutatum* Bösenberg & Strand, *Telamonia bifurcilinea* Bösenberg & Strand, and *Erigone sp.* as the dominant predatory spider species [54]. Moreover, the populations of *Erigone sp.* were higher in tea intercropped with *P. notatum*, while the absolute abundance of spiders was greater in tea intercropped with *C. rotundifolia* [54]. In tea plantations intercropped with *C. tora*, the predominant predatory jumping spider species were *Evarcha albaria* L. Koch and *Plexippus paykulli* Audouin, which preyed upon tea green leaf hopper (*E. onukii* Matsuda) [66]. Intercropping tea plants with citrus, waxberry, and snake gourd helped suppress tea green leafhopper (*E. vitis* Gothe), with the increased diversity of key araneid natural enemies [70]. Furthermore, population density and spatial distribution patterns of the leafhopper and the Orb-weaver spiders (araneids) were regulated, thereby checking the population abundance of tea leaf hopper (*Empoasca vitis* Gothe) [70]. 

#### 3.1.3. Mites

Diversifying tea agroecosystems using cover crops can strengthen predatory mite densities, thereby providing a viable biocontrol tool and promoting the environmentally benign production of tea products [43,74]. In tea plantations, mites constitute one of the most critical and complex communities of arthropods, where intercropping cover crops assist in augmenting the densities of the predatory Whirligig mite (*Anystis baccarum* L.) [75]. Tea plantations, where *P. notatum* and *C. rotundifolia* are used as intercrops, exhibited a high number of individuals (N), higher species richness (S), effective diversity index (eH’), and absolute abundance (n) of predatory *Anystis baccarum* L. [76]. Liu [71] mentioned that tea intercropped with *A. conyzoides* and *C. tora* saw a decrease in tea green leaf hopper (*E.pirisuga*) abundance due to the augmented density of *A. baccarum*. Furthermore, intercropping cover crops like *P. notatum* and *C. rotundifolia* helped to enhance the densities (per unit abundance) of the predatory mite, *A. baccarum*, in the tea canopies and may reduce the populations of the leafhopper pest, *E. onukii* [77].

#### 3.1.4. Lacewings

Lacewings, voracious predators, are predominantly seen in tea plantations and assist in biological control. Intercropping diverse cover crops in tea plantations increased the abundance of green lacewings, stimulating the biological control of tea pests. The diversity of predatory lacewings (*Chrysopa septempunctata* Wesmae1) was increased when *C. rotundifolia* and *I. hendecaphylla* were used as cover crops in tea plantations, which helped in the control of green leaf hoppers and tea geometrids [43]. Moreover, tea plants intercropped with *C. tora* demonstrated a marked increase in *Chrysopa sinica* Tjeder [66].

#### 3.1.5. Parasitoids

Parasitoids are essential ecosystem service providers renowned for their role as biocontrol agents in sustainable pest-management strategies [76]. Parasitoids can be used as biological control agents to mitigate pest attacks in tea agroecosystems. Zhang et al. [66] revealed that the critical parasitoid species in aromatic plants (*Leonurus Artemisia* Lour. and *C. tora*) intercropped in a tea garden were *Ephedrus plagiator* (Nees), *Amitus hesperidum* Silvestri, *Aphelinus mali* Haldeman, and *Apanteles adoxophyesi* Minamikawa. The most dominating parasitoids included families belonging to Eulophidae, Encyrtidae, Mymaridae, and Scelionidae, while abundant parasitoids included families belonging to Braconidae, Aphelinidae, and Trichogrammatidae in the tea plantation intercropped with cover crops (*C. rotundifolia*, *I. hendecaphylla*, and *V. sinensis*) [43]. The cover crops (*I. hendecaphylla* and *C. rotundifolia*) intercropped with tea constituted a higher species richness of parasitoids. *I. hendecaphylla* harbored more significant numbers of Braconidae, Eulophidae, and Aphelinidae parasitoids, while *C. rotundifolia* harbored Trichogrammatidae parasitoids [43]. Furthermore, Chen et al. [43] reported that the incorporation of *V. sinensis* as a cover crop in tea plantations increased the abundance of Eulophidae parasitoids. 

## 4. Cover Crops, Tea Yield, and Biological Control

The incorporation of leguminous cover crops (hairy vetch, soybeans, sunn hemp, and winter peas) can generate the following substantial input cost savings for cash crop. The leguminous covers help add and retrieve nutrients by providing (45–224 kg ha^−a^) of available N for cash crop production [77]. The cover crops promote long-term sustainability and higher cash-crop yields, with reduced fertilizer, herbicide, and pesticide applications [78]. The combined utilization of leguminous and grass cover crops has been reported for multiple ecosystem services (biological N_2_ fixation, weed control, attracting pollinators, and increasing SOM) [79]. Moreover, leguminous cover crops remarkably increased subsequent primary crop yields by 9.7% on average compared to fallow across China [80].

Soybean and tea cover cropping improved secondary metabolites with enhanced N uptake, encouraging the reduced application of synthetic nitrogen fertilizers [81]. Tea plants intercropped with white clovers (*T. repens*) saw an increase in tea yield by 32.6% and a decrease in the ratio of polyphenol to amino acid (TP/FAA) in spring tea and autumn tea (17.10% and 30.90%) [67]. Intercropping tea with *Vulpia myuros* L. improved tea root activity with accelerated nutrition uptake and increased functional tea quality components (free amino acids, polyphenols, and caffeine) [82]. Cover crops also have potential in pest management, as they can break pest cycles [83]. Intercropping cover crops in tea rows outranks tea rows clearance and natural weed cover, where enhanced tea production and biological pest control were considered the additional benefits of cover crops [67,84]. *Litsea cubeba* Lour, a vegetative branch inter-row cover, has been suggested as an important biological control agent in sporadic planting or branch coverage in tea plantations [85].

**Figure 3:**
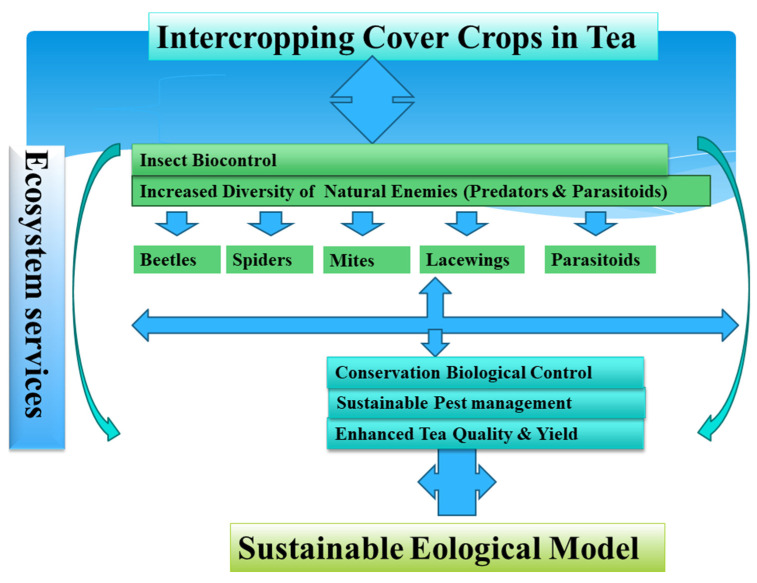
Ecosystem services provided by cover crop intercropping in the tea agroecosystem.

Despite the broad potential in agricultural pest management, cover crops are still not considered a silver bullet due to the risks associated with environmental stewardship [86]. The wide adoption of cover crops has not been achieved yet due to the economic, biological, and farm operational factors and uncertainties in pest dynamics [87]. The excessive use of pesticides might provide increased yield for a few years, but in the long run it will depopulate the beneficial arthropods [88]. Hence, cover crops will quantify ecosystem services, including increased yield, quality, and biological pest control in the tea agroecosystem (as seen in Figure 3). 

## 5. Conclusions and Further Recommendations

Tea, a woody perennial extensively cultivated across five continents in various agricultural systems, supports the daily livelihoods of resource-poor farmers and rejuvenates regional economies [89]. The tea ecosystem is a reservoir of resources and a specialized niche for more than 1000 arthropod species, including beneficial and pest species [27]. Intensive monoculture makes tea plantations vulnerable to insect pests and diseases due to the low diversity of natural enemies and the shift in soil microbial community structure and diversity [90]. The intercropping of cover crops appears to be a viable alternative for ecological intensification, delivering complete ecosystem services regarding pest regulation in the tea plantations. 

The selection of appropriate cover crop species promotes agrobiodiversity and, in the meantime, assists in quantifying ecosystem services in the tea plantations pushing towards a sustainable mode of tea production. Climate-resilient crops (sorghum, cowpea), volatile-emitting aromatic plants (semen cassiae, marigold, flemingia), and leguminous covers are recommended as the most effective cover crops in tea plantations. Leguminous cover crops fix atmospheric nitrogen; aromatic plant covers can attract and repel tea pests due to the emission of essential volatiles. Climate-resilient legumes (drought-resistant, allelopathic, and with a short life cycle) and cereal covers assist in natural pest suppression. In addition, these crop species attract diverse natural enemies (e.g., ladybird beetles, parasitoids, spiders) and help suppress key tea pests (tea green leaf hopper, mirid bug, white flies, and tea geometrid) with the conservation biological control approach.

Therefore, cover cropping can be a promising alternative strategy for ecological intensification in a tea agroecosystem, enhancing the diversity of natural enemies and promoting sustainable pest control. Despite the remarkable rise in tea science research regarding beneficial aspects of cover cropping, a thorough understanding of cover crops and insect–tea–natural enemy interaction is so far insignificant. This review provides a necessary first step towards in-depth future discourse on this topic. Persistent studies on intricate relationships between cover crops, natural enemies, and insect herbivores are required to strengthen ecosystem services’ delivery in the biocontrol of tea pests. 

## Figures and Tables

**Table 1:** Ecosystem services of cover crops and potential cover crops in the tea agroecosystem.

Cover Crops	Intercrops	Ecosystem Services	Cited References
**A.** **Cereals**			
Buckwheat [*Fagopyrum esculentum* Moench]	Potato, soybean, sunn hemp, coffee, squash, cotton	An intelligent cover crop that grows well in low-quality soil and retrieves P; Flowers provide pollen and nectar to pollinators (*Apis* sp. and *Bumbus* sp.); Attracts beneficial insects [vespids, syrphids, Wedge beetle (*Macrosiagon cruenta* Germar 1824)]; Nectar sources for the parasitoid wasp (*Microplitis croceipes* Cresson); Buckwheat + Squash reduced the number of white flies and aphids; Buckwheat + cotton increased parasitism of the Mirid bug *(Apolygus lucorum* Meyer-Dur).	[28,29,30,31,32]
Sorghum [*Sorghum bicolor* L. Moench]	Maize, pearl millet, castor, pigeon pea, cowpea, cluster bean, tea	Sorghum + tea helps in mitigating global warming and reducing N_2_O emissions; Sorghum as an intercrop reduced whiteflies’ attack in castor, and increased natural enemies (coccinellids, microplitis, and spiders); Parasitoids’ (*Telenomous* sp. and *Trichogramma* sp.) activity was higher on castor + cowpea+ black gram + sorghum.	[33,34,35]
**B.** **Legumes**			
Guar [*Cyamopsis tetragonoloba* L.]	Pearl millet, sesame, green gram, sorghum, maize	Drought-tolerant multipurpose industrial grain legume of the arid and semiarid regions; Allelopathic compounds (pyrrolizidine alkaloids) act as repellents for herbivorous pests and parasitic nematodes; Guar + pearl millet + cluster bean + sorghum + guar: low incidence of leaf hopper, whitefly, and cowpea aphid.	[36,37,38]
Cowpea [*Vigna* spp. L.]	Maize, sorghum, cotton, forage legumes, tea	Leguminous cover crops, mitigate climate-change impacts; Cowpea as an intercrop enhances ecosystem services by attracting pollinators (honey bees, bumble bees, carpenter bees, wasps, butterflies, and moths); Cowpea + cotton: control of thrips and whiteflies; *V. sinensis* + tea; abundant eulophidae parasitoids; French bean + cowpea + tea: increased tea yield.	[39,40,41,42,43]
Tephrosia [*Tephrosia vogelii* Hook F.]	Coffee, tea, rubber, coconut, maize, cassava, oil-palm, citrus	Traditional legume cover crop; fixes atmospheric nitrogen; Botanical pesticides from extracts (rotenone, tephrosin, and sesquiterpenes) of fish bean; controls insect pests (thrips, whiteflies, and aphids).	[44,45]
Hairy-indigo [*Indigofera hirsuta* L.]	Rubber, tea, coffee, citrus, sunn hemp	A summer cover crop; can cope with drought stress; Hairy indigo + sunn hemp reduced greenhouse whitefly abundance by increasing ladybeetle predators; Hairy indigo + pecan: attracts beneficial insects (lady beetles).	[46,47]
Sunn hemp [*Crotolaria juncea* L.]	Buckwheat, coffee	Excellent cover crop; provides floral resources for predators (Vespids); Sunn hemp + coffee + buckwheat: lower spider mite (Tetranychidae) population.	[26,48]
**C.** **Aromatic plants**			
Lavender [*Lavandula* spp.]	Tea	Lavender (*Lavandula pinnata* L.) grows well under tea-growing conditions; Lavender odor volatiles repelled tea geometrids (*Ectropis oblique* Prout) and green leaf hopper (*Empoasca vitis* Gothe); Lavender as an intercrop reduced the green leaf hopper (*Empoasca vitis* Gothe) population in the tea plantation.	[49,50]
Marigold [*Tagetes erecta* L.]	Flemingia, tea	Excellent source of semiochemicals: *Tagetes erecta* L. and *Flemingia macrophylla* (Willd.) Merr; Environmentally safe control strategy against the tea green leafhopper, *Empoasca flavescens* Fabricius; Intercropping *T. erecta* and *F. macrophylla* reduce the tea green leaf hopper population due to the attractant blend volatiles (cis-3-hexen-1-ol, cis-3-hexenyl acetate, nonanal, and α-farnesene).	[51]
Semen cassiae [*Cassia tora* L.]	Tea, mint, Chinese motherwort	Semen cassiae (*Cassia tora* L.) + tea + mint (*Mentha haplocalyx* Briq) + Chinese motherwort (*Leonurus artemisia* Loureiro): control of TGL (*Empoasca vitis* Gothe) due to the release of the functional plant volatiles (p-cymene, limonene, and 1,8-cineole); Increased number of natural enemies: spiders, coccinellids, and lacewings.	[52]
**D.** **Others:** Maize (*Zea mays* L.), Mountain pepper (*Litsea cubeba* Lour), White clover (*Trifolium repens* L.), Round-leaf cassia (*Chamaecrista rotundifolia* Greene), and Creeping indigo (*Indigofera hendecaphylla* Jacq.): all these cover crops helped in the suppression of crucial tea pests (TGL, geometrids, white flies, tea aphids) and the increase in the abundance of natural enemies (spiders, ladybird beetles, parasitoids).			

Note: ‘+’ indicates the intercropped crop species in the agroecosystems, ‘P’ indicates Phosphorous, N_2_O indicates nitrous oxide and TGL indicates tea green leaf hopper.
